# Strong inflammatory signatures in the neutrophils of PAMI syndrome

**DOI:** 10.3389/fimmu.2022.926087

**Published:** 2022-09-20

**Authors:** Wenjie Zheng, Xiaorui Fan, Zhaohui Yang, Yaoyao Shangguan, Taijie Jin, Yan Liu, Jiqian Huang, Xiaohua Ye, Qing Zhou, Xiaozhong Li

**Affiliations:** ^1^ Department of Nephrology and Immunology, Children’s Hospital of Soochow University, Suzhou, China; ^2^ Department of Pediatric Rheumatology, The Second Affiliated Hospital and Yuying Children’s Hospital of Wenzhou Medical University, Wenzhou, China; ^3^ Life Sciences Institute, Zhejiang University, Hangzhou, China; ^4^ Department of Rheumotology, Dalian Municipal Women and Children’s Medical Center, Dalian, China

**Keywords:** inflammation, neutrophil, PAMI syndrome, PSTPIP1, pyrin inflammasome

## Abstract

PSTPIP1 (proline-serine-threonine phosphatase-interactive protein 1)–associated myeloid-related proteinemia inflammatory (PAMI) syndrome is a rare autoinflammatory disease caused by heterozygous gain-of-function mutation in PSTPIP1. As one of the PSTPIP1-associated inflammatory diseases (PAIDs), neutropenia is a distinct manifestation to separate PAMI syndrome from other PAIDs. This study aimed to investigate the potential role of neutrophils and inflammatory signatures in the pathogenesis of PAMI. PAMI neutrophils displayed markedly increased production of interleukin-1β (IL-1β) and IL-18 by enzyme linked immunosorbent assay (ELISA) assay and intracellular cytokine staining. ASC speck formation and lactic dehydrogenase (LDH) release are also increased in patient neutrophils suggesting elevated pyrin inflammasome activation followed by upregulated cell death in PAMI neutrophils. RNA sequencing result showed strong inflammatory signals in both nuclear-factor kappa B (NF-κB) pathway and interferon (IFN) pathway in patient neutrophils. This study highlighted that elevated proinflammatory cytokines IL-1β and IL-18, increased pyrin inflammasome activation, and upregulation of NF-κB and IFN signaling pathways in neutrophils play important roles in pathogenicity of PAMI syndrome.

## Introduction

PSTPIP1 (proline-serine-threonine phosphatase-interactive protein 1)–associated myeloid-related proteinemia inflammatory (PAMI) syndrome is a rare disease combined autoinflammatory and immunodeficiency. It is caused by heterozygous mutation p.E250K or p.E257K in PSTPIP1 (1). PAMI syndrome shares three main clinical features with PAPA (pyogenic arthritis, pyoderma gangrenosum and acne) syndrome (1). Neutropenia, hepatosplenomegaly, high myeloid-related protein 8 (MRP8) and MRP12 concentration are distinct manifestation to separate PAMI syndrome from PAPA syndrome, which is also caused by PSTPIP1 mutation (1).

PSTPIP1 is a cytoskeleton-associated adaptor protein involved in regulation of the actin cytoskeleton. It could bind with pyrin and promote the interaction of pyrin and apoptosis-associated speck-like protein containing a CARD (ASC) to facilitate pyrin inflammasome formation (2, 3). Compared with p.E250Q, the most common mutation in PAPA syndrome, p.E250K or p.E257K mutation of PSTPIP1, shows increased interaction with pyrin due to charge reversal in the y-domain (1). As a consequence, pyrin inflammasome was assembled to activate caspase-1 and process proinflammatory cytokines pro–interleukin-1β (IL-1β) and pro–IL-18 into mature IL-1β and IL-18 (4–6).

IL-18 is expressed by epithelia cells and macrophages. It has an endogenous antagonist called IL-18 binding protein that is induced by IFN-γ. Although serum IL-18 elevation has been previously reported associated with macrophage activation syndrome (MAS) in systemic juvenile idiopathic arthritis, adult-onset Still’s disease, and NLR family CARD domain containing 4 (NLRC4) inflammasomopathy autoinflammation (7, 8), recent study revealed that PAPA syndrome is associated with chronic and unopposed elevation of serum IL-18 levels without risk of MAS (9). Studies have shown that neutrophils play a potential pathogenic role in PAPA syndrome. Enhanced neutrophil extracellular trap formation has been detected in neutrophils, low-density granulocytes, and skin biopsies of patients with PAPA (10), which was related to the elevated inflammatory cytokines. Although caused by mutations in the same gene, PAMI syndrome is a more refractory autoinflammatory disease compared with PAPA syndrome, and the disease mechanisms of PAMI syndrome are still poorly understood. This study aimed to investigate the potential role of neutrophils and inflammatory signatures in the pathogenesis of PAMI syndrome.

## Materials and methods

### Patient and sample

Patient and patient control (P2) were recruited under protocols approved by the Institutional Review Board and the Medical Ethics Committee of The Second Affiliated Hospital and Yuying Children’s Hospital of Wenzhou Medical University (number 2021-K-349-02). The parents of the patients provided written informed consent. Patient control (P2) was a 9-year-old girl who presented with anemia at the age of 3 years and then developed with skin ulceration, pancytopenia, and splenomegaly. Whole exome sequencing (WES) revealed a *de novo* heterozygous c.748G>A, p.E250K, pathogenic variant in the PSTPIP1 gene, so PAMI was diagnosed. Her condition was partially controlled with steroid and cyclosporine. All of the following laboratory investigations in neutrophils were performed after immunosuppressive therapy.

### Whole exome sequencing

DNA from whole blood was extracted using the Maxwell RSC Whole Blood DNA Kit (Promega, AS1520). One microgram of DNA was used for WES. WES and data analysis were performed as previously described (11–13). The identified variant was then confirmed by Sanger sequencing.

### Cell preparation

Peripheral blood mononuclear cell (PBMCs) and neutrophils were separated by lymphocyte separation medium (LSM) and dextran according to the manufacturer’s instructions, respectively. PBMCs and neutrophils were grown in RPMI-1640 (Gibco) supplemented with 10% fatal bovine serun (FBS) and penicillin/streptomycin.

### Intracellular cytokine staining

Intracellular cytokine staining for IL-1β was measured in neutrophils at baseline. Cells were washed twice with phosphate belanced solution (PBS), then treated with Golgi plug (BD Biosciences) for 6 h at 37°C, with 5% CO_2_, and then permeabilized with Perm/Fix for 30 min at 4°C. Cells were stained by antibodies IL-1β (BioLegend, cat. no. 508208). All events were acquired on BD LSRFortessa (BD Biosciences) and analyzed by FlowJo (TreeStar).

### ASC speck formation

Neutrophils (5 × 10^4^) of patient were seeded on each well of 24-well plate with one poly-L-lysine–coated 12-mm glass coverslip (Shanghai Jing An Biology, J24002). The plate was incubated in a humidified incubator (37°C, 5% CO_2_) for 3 h. The coverslips were fixed with 500 μl of 4% Paraformaldehyde (PFA) (15 min, 37°C) and rinsed three times with 1× PBS. The coverslips were blocked with blocking buffer (1× PBS/5% normal serum/0.3% Triton™ X-100) for 60 min. The coverslips were incubated with the diluted primary antibody [ASC/TMS1 (E1E3I) rabbit mAb, #13833] overnight at 4°C and then were rinsed with the 1 × PBS. The coverslips were incubated with diluted fluorochrome-conjugated secondary antibody for 1–2 h at room temperature in dark and rinsed with 1× PBS. Nuclei were stained with 4,6-diamino-2-phenyl indole (DAPI) for 10 min. The coverslips were visualized using zeiss LSM 710 laser scanning confocal microscope, and images were acquired using ZEN-Blue. Fiji-ImageJ software was used to analyze the images. The percentage of ASC-specks containing cells was calculated as the fraction of ASC-positive specks containing specks.

### Cytokine measurement and LDH detection

Cytokine concentrations for IL-1β and IL-18 in serum were measured by Human IL-1β/IL-1F2 Duoset ELISA (R&D, DY201) and Human Total IL-18 Duoset ELISA (R&D, DY318) according to the manufacturer’s instructions. LDH released in supernatant was measured with the LDH Cytotoxicity Assay Kit (Beyotime, C0017) according to the manufacturer’s instructions. The concentrations of IL-6 in serum were measured by the BD Cytometric Bead Array (BD FACSCanto) according to manufacturer’s instructions (P010001-111). Data were analyzed by FCAP (3.0.1) software (BD FACSCanto).

### RNA sequencing

RNA libraries were generated using the NEBNext Ultra RNA Library Prep Kit for Illumina (New England Biolabs) and then sequenced on Illumina NovaSeq to get 150–base pair paired-end reads. featureCounts was used to count the reads numbers mapped to each gene. Differential expression analysis was performed using the DESeq2 R package.

## Results

**Figure 1:**
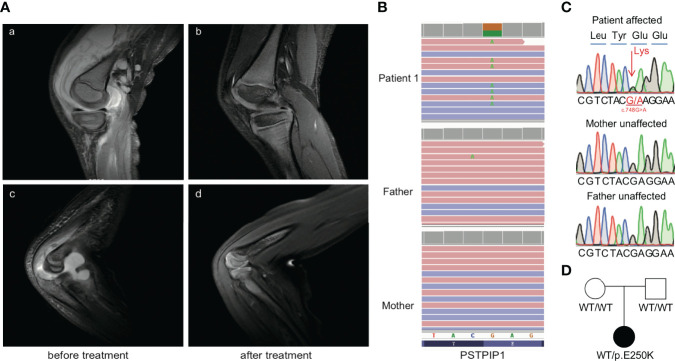
Clinical manifestation of pre- and post-treatment and confirmation of the PSTPIP1 mutation in the patient with PAMI. **(A)** T2WI MRI signals of knee and elbow joints pretreatment **(A, C)** and post-treatment **(B, D)**. **(B)** Exome sequencing reads covering the p.E250K variant in patient, displayed by the integrative genomics viewer. **(C)** Sanger sequencing confirmed the PSTPIP1 p.E250K variant. **(D)** Pedigree of the family with p.E250K variant in PSTPIP1.

A 6-year-old female patient born to Chinese healthy parents presented with mild to moderate anemia (HGB, 80–100 g/L) from the age of 6 months. At 1 year old, she presented with swelling and pain of the left ankle, fever, and hepatosplenomegaly accompanied with notable increase in inflammatory indexes. Joint incision and drainage was done as pyogenic arthritis in local hospital. Then, she developed hypotension and pancytopenia after operation. Serum ferritin was high at 1,634 ng/ml. Laboratory investigation showed elevation of liver enzymes (alanine transaminase, 151 U/L; aspartate aminotransferase, 312 U/L), hypertriglyceridemia (2.2 6 mmol/L), and hypofibrinogenemia (0.85 g/L). A small number of phagocytes were found in bone marrow cytology, and bone marrow biopsy revealed myelofibrosis. She was diagnosed with septic shock and treated with imipenem, vancomycin, and high-dose dexamethasone (about 1.1 mg/kg/day). Her condition was relieved, and dexamethasone and antibiotic were withdrawn in 3 weeks. At the age of 4 years, she was hospitalized because of fever and swelling and pain of her right elbow and left knee joint. Joint fluid routine revealed white blood cell count (WBC; 100–150 ×10^9^/L) with > 90% neutrophils but negative gram stain and culture. Joint ultrasound showed thickened synovium and joint effusion of elbow and knee joint. Magnetic resonance imaging (MRI) of the elbow and knee indicated diffuse bone marrow edema, thickened synovium, and massive joint effusion (**Figure 1A**). Laboratory testing showed elevated WBC (10.44 × 10^9^/L), absolute neutrophil count (ANC; 3.8 × 10^9^/L), platelet count (690 × 10^9^/L), decreased hemoglobin (69 g/L), as well as increased acute phase reactants such as serum amyloid A (SAA; 152 mg/L), C-reactive protein (CRP; 173 mg/L), and erythrocyte sedimentation rate (ESR, 64 mm/h). Ibuprofen was initiated with the dose of 28.6 mg/kg/day. One week later, ESR and CRP decreased to 38 mm/h and 31 mg/L, respectively. However, leukopenia and neutropenia (WBC, 2.88×10^9^/L; ANC, 0.67×10^9^/L) were observed while the inflammatory episode was partially controlled.

Considering the recurrence of the disease, autoinflammatory disease was suspected. Prednisone tablets (about 0.71 mg/kg/day) combined with IL-6 inhibitor tocilizumab of 160 mg (11.4 mg/kg) every 4 weeks were initiated, but ESR, CRP, and SAA were not controlled well. Then recombinant human tumor necrosis factor–a (TNF-a) receptor fusion protein etanercept(0.8 mg/kg every week), cyclosporine (3 mg/kg/day) combined with methotrexate (9.8 mg/m2)were initiated, and the disease was controlled efficiently. ESR, CRP, and SAA gradually returned to normal, and hepatosplenomegaly gradually subsided. Reexamination of the left knee and right elbow joint MRI indicated that the effusion in the articular cavity was obviously reduced and synovitis was significantly improved (**Figure 1A**). Without arthritis and fever, the remission state had been maintained for a year until now, and the amount of prednisone has been reduced to 5 mg daily (about 0.26 mg/kg/day). No pyoderma gangrenosum and acne were found during follow-up. Neutropenia persists despite being inactive status (minimum, 0.61 × 10^9^/L), but no recurrent infections were observed. The genotype was a de novo mutation of p.E250K(c.748G>A) in the PSTPIP1 gene and was also validated by Sanger sequencing (**Figures 1B–D**), so the diagnosis of PAMI syndrome was confirmed. 

**Figure 2:**
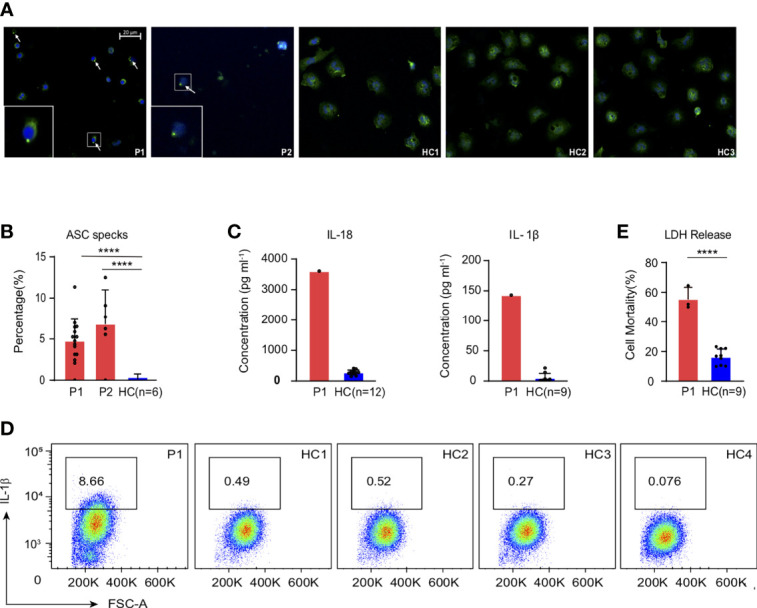
Strong inflammatory signatures in the patient with PAMI compared with healthy controls. **(A, B)** Increased ASC speck formation in the neutrophils of the patients (P1 and P2) compared with six healthy controls. **(C)** Excess levels of IL-18 and IL-1β in the serum of the patient1 compared with 12 and 9 healthy controls, respectively. **(D)** Higher IL-1β level in the neutrophils of the patient1 compared with four healthy controls. **(E)** LDH release in the neutrophils of the patient1 compared with three healthy controls. ****p<0.0001.

Neutropenia is a common symptom in PAMI syndrome, and our patient showed low ANC as well. The reduction of neutrophil happened after inflammation, but the relationship between inflammation and neutropenia is still unclear in patients with PAMI syndrome. We first measured ASC speck formation, the hallmark of pyrin inflammasome activation (14, 15), in the patient neutrophils. We observed higher ASC speck compared with healthy controls (**Figures 2A, B**); the similar level of increased ASC specks was also confirmed in the second patient with PAMI syndrome (P2), which indicated increased pyrin inflammasome assembly and activation in neutrophils of the patients with PAMI. Then, we measured IL-1β and IL-18 concentration released in serum by ELISA assay, and the patient showed significant increased cytokines compared with healthy controls (**Figure 2C**). In addition, the results were consistent with the excess serum IL-18 observed in patients with PAPA (9), and it also established the association of IL-18 with PAMI syndrome. To confirm whether neutrophils contributed the cytokine release in serum, we analyzed the IL-1β production in neutrophils with intracellular cytokine staining. We detected elevated level of IL-1β in patient neutrophils at basal level compared with four healthy controls (**Figure 2D**). The excess production of IL-1β in neutrophils of the patient with PAMI further confirmed that pyrin inflammasome was over activated to promote downstream inflammatory cascade reaction in the patient.

Moreover, the activation of pyrin is one of the prerequisites for triggering pyroptotic cell death (pyroptosis) (16, 17). Activated caspase-1 can specifically cleave Gasdermin D (GSDMD). The N-terminal of GSDMD was released to form pores in cell membrane for cell contents release and cell rupture and leads to inflammation (18–20). We measured LDH release, a cytoplasmic enzyme released by dying cells that lose cell membrane integrity (21), and increased LDH protein could be detected in culture supernatant of patient neutrophils at the basal level (**Figure 2E**). The results indicated increased cell death in the neutrophils of the patient with PAMI promoted by pyrin inflammasome activation.

**Figure 3:**
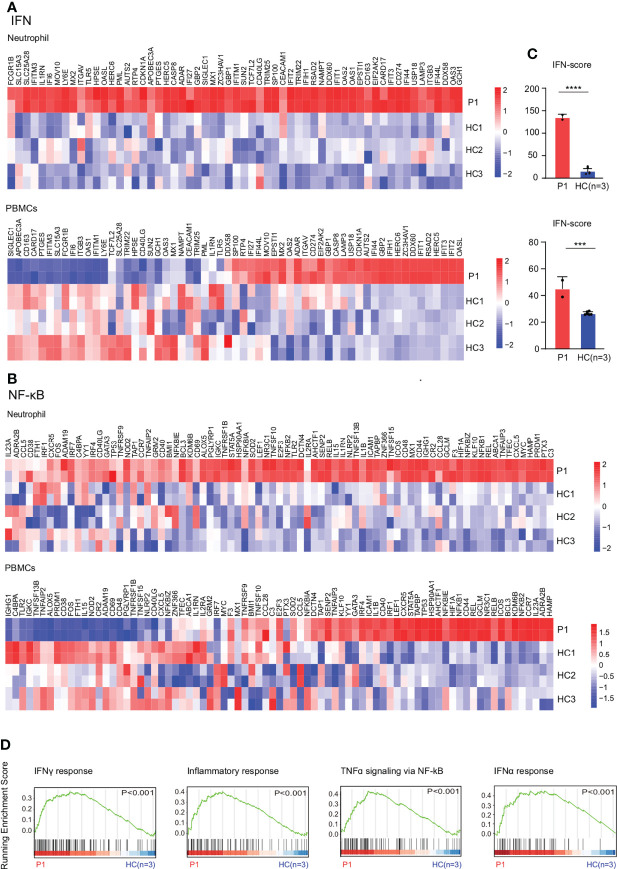
Expression patterns of genes involved in IFN and NF-κB by RNA sequencing. **(A)** Upregulation of IFN pathway in neutrophils and PBMCs of the patient1 compared with three healthy controls. **(B)** Upregulation of NF-κB pathway in neutrophils and PBMCs of the patient1 compared with three healthy controls. **(C)** IFN response gene scores (28 genes) in neutrophils and PBMCs of the patient1 and healthy controls, determined with RNA sequencing data. **(D)** GSEA plot of IFN-γ response, inflammatory response, tumor necrosis factor–α (TNF-α) signaling *via* NF-κB, and IFNα response in neutrophils from the patient1 and controls. ****p<0.0001. ***p<0.001.

RNA sequencing results showed upregulation of NF-κB and IFN signaling pathways in the patient’s neutrophils and PBMCs compared with healthy controls (**Figures 3A, B**). The results were confirmed by higher IFN scores in the patient with PAMI compared with healthy controls (**Figure 3C**). In addition, the inflammatory gene expression is more elevated in neutrophil than in PBMCs, and the IFN score is also higher in neutrophil than in PBMCs in the patient. Therefore, the inflammatory signature is higher in neutrophils compared with PBMCs in the patient with PAMI (**Figures 3A–C**). The Gene Set Enrichment Analysis (GSEA) plot showed higher IFN-γ pathway, inflammatory response, TNF-α signaling *via* NF-κB, and IFNα pathway in patient neutrophils than controls (**Figure 3D**). Consistent with NF-κB pathway activation, the patient showed elevated IL-6 level of 120.5 pg/ml (normal range, 1.7–16 pg/ml) in serum.

## Discussion

The main hematologic manifestation in PAMI syndrome is neutropenia, but pancytopenia is also observed in a few cases in acute active phase just like our patient in this study (22, 23). In our patient, serum ferritin was high accompanied with hypertriglyceridemia and hypofibrinogenemia, so it fulfilled the criteria of MAS but misdiagnosed as septic shock at that time. MAS has been reported in the previous literature (24), and we speculate that the operation may be the stimulating factor of MAS in our patient, because the recurrence of MAS was not observed in the subsequent acute active phase. Bone marrow biopsy in this case showed myelofibrosis. Although myelofibrosis was not a common finding and the specific pathogenesis is not clear, it had also been reported in other patients with PAMI (25, 26).

Our study showed that the patient with PAMI exhibited strong inflammatory signals in neutrophils including over production of proinflammatory cytokines IL-1β and IL-18, hyperactivation of pyrin inflammasome, and excess cell death and upregulation of NF-κB and IFN signaling pathways, which resulted in severe inflammation and may contribute to develop neutropenia in patients with PAMI syndrome.

Patients with PAMI syndrome were rarer than PAPA syndrome, and some cases remain refractory to treatment, suggesting that the disease is more difficult to be controlled. hematopoietic stem cell transplant (HSCT) has been proved as effective treatment for patients with PAMI syndrome (24). According to previous reports, IL-1 inhibition did not resolve neutropenia in PAMI syndrome (1, 25), whereas it was effectively used for pyrin inflammasome–associated inflammation control in other autoinflammatory disorders (27, 28). Targeting IL-1 combination with IL-18 inhibition might be helpful for disorders with overproduction of both IL-1β and IL-18. Our study identified hyperactivation of inflammatory signals in both NF-κB and IFN pathways in the patient. In addition, the patient responded well to TNF inhibitor, etanercept, and cyclosporine combined with steroid and methotrexate, suggesting that targeting NF-κB signaling pathway could also help to suppress the inflammation in PAMI syndrome. Nevertheless, this study has certain limitations. First, immunosuppressive therapy implemented in the patient could influence the results. Second, more patients’ samples are needed to provide a comprehensive analysis of the inflammatory signature of PAMI syndrome, including a comparison of inflammatory patterns of neutrophils with macrophages or monocytes of PAMI syndrome and with neutrophils of PAPA syndrome. The inflammatory signatures in neutrophils revealed in the patient with PAMI in our study provided insights for better understanding of the disease mechanisms.

## Data availability statement

The original contributions presented in the study are included in the article/supplementary material, further inquiries can be directed to the corresponding author/s.

## Ethics statement

The studies involving human participants were reviewed and approved by The Institutional Review Board and the Medical Ethics Committee of Second Affiliated Hospital and Yuying Children’s Hospital of Wenzhou Medical University (number 2021-K-349-02). The patients/participants provided their written informed consent to participate in this study.

## Author contributions

QZ and XL designed the study and directed and supervised the research. WZ, ZY, TJ, and XF performed experiments and analyzed the data. WZ, YS, YL, JH, and XY enrolled the patients and collected and interpreted clinical information. WZ, XF, TJ, QZ, and XL wrote the manuscript with input from other authors. All authors contributed to the review and approval of the manuscript.

## Funding

This work was supported by the National Natural Science Foundation of China (NSFC81370787, NSFC31771548, and NSFC 81971528), Zhejiang Provincial Natural Science Foundation of China (LR19H100001), Zhejiang Provincial Health Science and Technology Project (2022KY904), Suzhou Key Discipline of Pediatric Immunization (szxk 202106), and Suzhou Civil Biotechnology Key Technology Project (ss02067).

## Acknowledgments

We thank the patient and healthy controls for support and help.

## Conflict of interest

The authors declare that the research was conducted in the absence of any commercial or financial relationships that could be construed as a potential conflict of interest.

## Publisher’s note

All claims expressed in this article are solely those of the authors and do not necessarily represent those of their affiliated organizations, or those of the publisher, the editors and the reviewers. Any product that may be evaluated in this article, or claim that may be made by its manufacturer, is not guaranteed or endorsed by the publisher.
